# CONTINUITY OF CAREER OVER THE LIFE COURSE ON LIFE SATISFACTION AND DEPRESSIVE SYMPTOMS IN LATER LIFE

**DOI:** 10.1093/geroni/igz038.468

**Published:** 2019-11-08

**Authors:** Melanie S Hill, James E Hill, Stephanie Richardson, Jessica Brown, Jeremy B Yorgason, Jeff Hill

**Affiliations:** 1 Brigham Young University, Provo, Utah, United States; 2 University of Utah, Salt Lake City, Utah, United States

## Abstract

Identity scholars have suggested that having a unified sense of past, present, and future is related to positive well-being outcomes (Whitbourne, Sneed & Skultety, 2009). One’s occupation can have a profound influence on an individual’s identity throughout the life course (Nazar & van der Heijden, 2012). Research has looked at career mobility among younger age groups (Baiyun, Ramkissoon, Greenwood, & Hoyte, 2018); however, less is known about the impact of career stability later in life. Consistency in career choice over the life course may have positive outcomes down the line as career becomes part of an individual's identity. The current study uses the Life and Family Legacies dataset, a longitudinal state-representative sample of 3,348, to examine individual’s careers at three points in the life course: high school (projected career choice), early adulthood, and later life. Results revealed that a match of desired career in high school and actual career in early adulthood was not predictive of life satisfaction or depressive symptoms in later life. However, a match of career in early adulthood and later life was significantly related to better life satisfaction and less depressive symptoms, which was explained through higher levels of job satisfaction. This study highlights the importance of acquiring and maintaining a career that is fulfilling to the individual over the course of early adulthood to later life.

